# Chronic Recanalization of Dissection of the Distal Anterior Cerebral Artery: Case Report and Review of the Literature

**DOI:** 10.1155/2009/303695

**Published:** 2009-08-20

**Authors:** Shuichiro Asano, Tetsuo Hara

**Affiliations:** ^1^Department of Neurosurgery, Toyama Hospital, International Medical Center of Japan, Tokyo 162-8655, Japan; ^2^Department of Neurosurgery, Tokyo Kosei-Nenkin Hospital, 5-1 Tsukudo-cho, Shinjuku-ku, Tokyo 162-8543, Japan

## Abstract

The natural history of atraumatic idiopathic dissection of the distal anterior cerebral artery is still unclear. We present a 38-year-old man who had dissection of the left A_2_ segment of this vessel associated with subintimal hematoma and infarction. Because of complete stroke in acute stage, he did not undergo surgery. About three months later, administration of aspirin (100 mg/day) was started. At nine months, magnetic resonance angiography revealed complete recanalization of the A_2_ dissection. To assess the outcome of dissection, we should observe the patient for at least one year.

## 1. Introduction

Atraumatic intracranial dissecting aneurysm mainly occurs in the vertebrobasilar or internal carotid arteries [1]. Although reports about dissecting aneurysms of the distal anterior cerebral artery have been gradually increasing, especially in Japan, the clinical course and treatment of this lesion are still unclear. We report an unusual case of dissection of the distal anterior cerebral artery that showed angiographic recovery by 9 months after the onset with antiplatelet therapy alone. We also review the relevant literature.

## 2. Case Report

**Figure 1:**
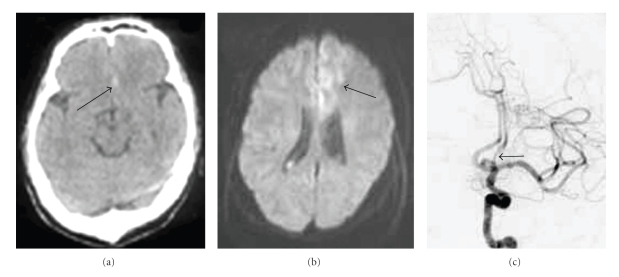
Imaging findings on the day of onset. (a) Computed tomography shows very thin subarachnoid hemorrhage on the left side of the interhemispheric fissure (arrow). (b) Magnetic resonance imaging (diffusion image) reveals infarction of the left medial frontal region (arrow). (c) Left internal cerebral artery angiography reveals stenosis (string sign) of the A_2_ segment (arrow).

While urinating, a 38-year-old man suffered from the sudden onset of severe headache, difficulty speaking, and right hemiparesis (the lower extremity was more severely affected). He was taken to a local hospital by ambulance. Computed tomography (CT) showed thin subarachnoid hemorrhage (SAH) on the left side of the interhemispheric fissure (Fisher's group 2 [2]) (Figure 1(a)). Magnetic resonance imaging (MRI) revealed left frontal lobe infarction (Figure 1(b)), and cerebral angiography indicated stenosis (string sign) of the distal left anterior cerebral artery (A_2_ segment) (Figure 1(c)). A tentative diagnosis of idiopathic SAH with early spasm of the left A_2_ segment was made. After two weeks of medical treatment, repeat cerebral angiography showed no source of SAH and persistent A_2_ stenosis. His speech improved, but right hemiparesis persisted. After transfer to a rehabilitation hospital, it was decided to re-examine his condition and he was admitted to our hospital.

**Figure 2:**
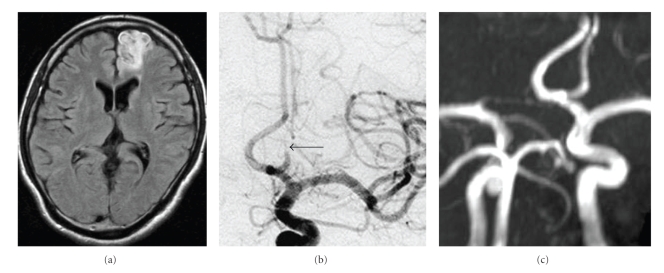
(a) Magnetic resonance imaging (FLAIR sequence) about one month after the onset shows infarction of the medial part of the left frontal lobe. (b) Left internal cerebral artery angiogram displays severe stenosis of the A_2_ segment (arrow). (c) About 9 months after the onset, magnetic resonance angiography reveals recanalization of the left A_2_ segment.

 On admission, he had right hemiparesis (4/5 for the upper limb on manual muscle testing and 2/5 for the lower limb). MRI showed medial left frontal lobe infarction with poor perfusion of the left A_2_ segment (Figure 2(a)). Angiography also revealed severe stenosis (string sign) of the left A_2_ segment (Figure 2(b)). Because the string sign without pear appearance (stenosis without dilation) warranted the diagnosis of the dissecting aneurysm by some articles [3–5], the final diagnosis was dissection of the left A_2_ segment of the anterior cerebral artery. Because his clinical status was stable, he was returned to the rehabilitation hospital. 

 Two months later, he was reviewed at our outpatient clinic. His right upper limb was recovered but his right lower limb was 3+ ~ 4/5 on manual muscle testing. Because the left distal anterior cerebral artery did not recanalize at that time, aspirin (100 mg/day) was commenced. Six months later, magnetic resonance angiography (MRA) showed recanalization of the left A_2_ segment (Figure 2(c)). Because repeat MRA also showed patency of the left A_2_ segment one year later, aspirin therapy was ceased. There have been no new neurological problems during followup.

## 3. Discussion

The present patient had a dissection of the distal anterior cerebral artery that showed angiographic recanalization in the chronic stage with antiplatelet therapy alone.

 We identified 42 detailed reports of atraumatic distal anterior cerebral artery dissection in patients without collagen diseases (including our case, Table 1) [6–40]. Their mean age was 47.3 years (SD 8.5), and the female:male ratio was approximately 1/4 (8 women [6–13], and 34 men [6, 14–40]). Only one patient was reported from outside Japan [7]. There were 30 cases of cerebral infarction alone [6, 8, 12–34], six cases of hemorrhage alone [7, 9, 14, 35–37], and six cases of both infarction and hemorrhage (including our case) [10, 11, 38–40]. In the infarction only group, 20 patients did not have surgery [6, 8, 12, 14–27], and the other 10 underwent surgical intervention (wrapping in two cases [15, 32], A_3_-A_3_ bypass and trapping in five cases [28–31], and trapping only in three cases [13, 33, 34]). Among the patients with infarction alone and conservative treatment, only three cases of angiographic improvement were reported [14, 16, 17]. In one of these three patients, urokinase (180,000 units/day) was administered for one week after infarction [16], antihypertensive and antiplatelet therapies were administered for six months in another patient [17], and ticlopidine (200 mg/day) was administered for 8 months to the third patient [14]. The period until angiographic recovery was three months, six months, and 8 months, respectively. 

 The patients who had hemorrhage alone included four with intracerebral hemorrhage (ICH) [7, 9, 35, 36], and two with SAH [14, 37]. All four patients with ICH had dissection of the A_4_ segment. Both SAH patients had dissection of the A_2_ segment, but they were neurologically normal while the other patients suffered from infarction and hemiparesis. A_3_-A_3_ bypass and trapping was done in one SAH patient [37], and wrapping was performed for the other [14]. 

 The combined hemorrhage and infarction group comprised five patients (including ours) with SAH and infarction [10, 11, 38, 39], as well as one patient with ICH and infarction who did not undergo surgery [40]. A_3_-A_3_ bypass and trapping was done in one patient with SAH and infarction [39], wrapping was performed in one [38], and three patients (including ours) were managed conservatively [10, 11]. Among the last three patients, recanalization of the dissection was confirmed in two, with one receiving no antiplatelet or anticoagulant therapy [10] and the other being our patient. The period from the onset until angiographic confirmation of recanalization was 18 months and 9 months, respectively. 

 We found five patients with infarction (including our case) who had confirmed recanalization of the dissection after conservative treatment [10, 14, 16, 17], while the dissecting aneurysm persisted in 19 patients [6, 8, 11, 12, 14, 15, 18–27, 40]. The median period until angiographic recanalization was 8 months (25th–75th percentile: 5.3–11.3 months; range: 3–18 months), while the median period until final angiography in the other 19 patients was only two months (25th–75th percentile: 1.5–4.0 months; range: 0.7–96 months) and the difference was significant (*P* = .029, Mann-Whitney *U*-test). Therefore, the angiographic cure rate of distal anterior cerebral artery dissection after conservative treatment may be increased by continuing followup for at least one year. 

 Although anticoagulation and/or antiplatelet medication may be a widely accepted conservative management for intracranial arterial dissection with purely ischemic symptom, two patients with infarction onset died of adverse drug reactions. One was administered sodium ozagrel (80 mg/day) for 12 days after infarction, but severe SAH occurred on the 12th day [13]. Another patient received antithrombin therapy in the acute stage, followed by an antiplatelet drug. On day 21 after the onset, he suffered from severe ICH [6]. On the other hand, 11 patients were safely administered urokinase [16], ozagrel [11, 18–20], heparin [21, 22], argatroban [12], ticlopidine [14], aspirin [12, 14, 21], or antiplatelet drugs [17] during the acute stage. Three of these 11 patients achieved angiographic cure [14, 16, 17]. These results suggest that we must pay close attention when using these drugs in the acute stage of distal anterior cerebral artery dissection.

## 4. Conclusion

We reported a patient with dissection of the anterior cerebral artery (A_2_ segment) who showed recanalization after 9 months with only aspirin therapy. It may be important to monitor such patients for at least one year to determine the clinical outcome.

## Figures and Tables

**Table 1:** Items of the atraumatic distal anterior cerebral artery dissection with 42 detailed cases reports (including our case).

	Conservative treatment group	Conservative treatment group	Surgical intervention group	Surgical intervention group	Surgical intervention group
	Recanalized	Not recanalized	Wrapping	A_3_-A_3_ bypass & trapping	Trapping
Infarction onset	3	17	2	5	3
SAH with A_2_ dissection onset	0	0	1	1	0
ICH with A_4_ dissection onset	0	4	0	0	0
Infarction with SAH onset	2	1	1	1	0
Infarction with ICH onset	0	1	0	0	0

SAH: subarachnoid hemorrhage, ICH: intracerebral hemorrhage.
